# Association of sexual perceptions, behavior, and intimate partner violence with sexually transmitted infection (STI) among Filipino women

**DOI:** 10.1186/s12889-025-25019-7

**Published:** 2025-11-04

**Authors:** Sophia Palma, Róbert Bata

**Affiliations:** 1 Faculty of Health Sciences, University of Debrecen, Debrecen, Hungary; 2 Faculty of Health Sciences, Department of Epidemiology, University of Debrecen, Debrecen, Hungary

**Keywords:** STI, Sexually transmitted infections, Domestic violence perception, Domestic violence, IPV, Sexual behavior, DHS, Philippines, Reproductive coercion, Women’s reproductive health

## Abstract

**Background:**

Sexually transmitted infections (STI) remain prevalent in the Philippines despite being both preventable and treatable. Women are particularly vulnerable when less prioritized than high-risk groups and unable to speak up in abusive intimate relationships. Although studies on behavior and STI have been conducted, they vary by culture and region, highlighting the importance of representative studies. This study aimed to identify sexual perceptions and practices, and intimate partner dynamics associated with STI in Filipino women.

**Methods:**

A secondary analysis of the 2022 Philippine Demographic Health Survey was conducted: 19,228 sexually active women in relationships, weighted by region. The outcome: a composite of self-reported STI and related symptoms in the last 12 months. Variables tested: sociodemographic factors, safe sex practices, and perceptions, such as the ability to refuse sex and ask a partner to wear a condom, justifying partner violence, and intimate partner coercion and abuse. Descriptive statistics and modified Poisson regression analysis were conducted to identify significant factors and estimate their risk ratios; the *p*-value (*p*) was set at < 0.05.

**Results:**

One thousand three hundred thirty-nine females reported having an STI (6.96% [CI: 6.19%, 7.73%]). Reproductive coercion nearly doubled the risk of STI. Women who perceived domestic abuse to be justified increased their risk of STI by 12%. Emotional violence and fear of one’s partner consistently increased the risk for STI (ARR: 1.29 and 1.33, respectively). Physical and sexual abuse were also associated with STI.

**Conclusion:**

Routine STI testing of IPV victims is recommended as well as expanding STI risk assessment to sexual perceptions and history of IPV. STI surveillance is incomplete when focused on high-risk groups only. Human rights-based approach on sexual practices, and gender equality should be values instilled in mandatory pre-marriage family planning seminars and sex education, grounded on mutual respect, and consent. Without correcting harmful perceptions on domestic abuse and IPV prevention, STI control may not be completely achieved.

## Introduction

The World Health Organization estimates over a million preventable STI cases daily among adults 15–49 years old [1]. In 2021, the prevalence of non-HIV STI among Southeast Asian women (22.68%) was higher than the overall regional average; Filipino women experience higher prevalence and incidence rates than the national figures, with no decline observed in the past five years [2]. STI disproportionately affect women younger than 25 years old, from low to middle-income countries (LMICs) [3].

Condom use and the number of sexual partners are known to influence STI prevalence. However, various underlying factors also shape these behaviors. Without accounting for them, infection control can be undermined. Individual factors such as substance and alcohol use can increase risky sex behavior [4–6]. In intimate relationships, communicating expectations of safe sex practices can protect women from potential STI risk through self-assertion [7–9]. In culturally conservative societies, education on safe sex practices and STI is often absent in classrooms when abstinence is promoted as the sole approach [6, 10, 11]. Intimate partner violence (IPV) is strongly linked to STI, particularly in young women with lower levels of education [12]. Coercive, unequal relationship dynamics and threatening environments contribute to women’s susceptibility to the infection [13]. However, social determinants differ depending on ethnicity and region: in Africa, higher education and wealth indices increased the odds of STI, while opposite findings were seen among American women [7, 14, 15]. This emphasizes the importance of representative studies to guide surveillance, prevention, and counseling programs.

STI surveillance in developing countries focuses on high-risk populations: female sex workers (FSW) and males having sex with males (MSM) [16–18]. Non-HIV STI and the general female population tend to be overlooked, leading to gaps in understanding the burden of infection. In the Philippines, traditional roles prevail where men are less likely to participate in reproductive health initiatives, believing it is beneath them as heads of households [19]. This puts female partners at risk of STI and other diseases. Hostility and altercations between couples were reported when condom use was brought up by the health centers [19].

To the best of our knowledge, this is the first study investigating sexual perceptions, practices, and domestic violence in women with STI using the 2022 P-DHS. Despite limitations of self-reporting, a nationally representative sample can shed light on STI susceptibility among women. The study aimed to determine the characteristics of sexually active Filipino women associated with having an STI: their sexual perceptions, behavior, and intimate partner dynamics, and to identify potential risk factors.

## Methodology

### Philippine demographic health survey

The dataset is available online upon request at the DHS website^1^. The Institutional Review Board of the Intercity Fund (ICF) reviewed and approved the study protocol; the Philippine Statistics Authority approved data collection and public use of datasets, assured anonymity, and secured consent of respondents [20]. The P-DHS employed a two-stage stratified sample design, utilizing 1247 primary sampling units drawn from a master sample frame (systematically selected from 33 highly urbanized cities and 42036 barangays). From these primary sampling units, 22 or 29 sampling housing units were randomly selected. In each housing unit, no more than three households were interviewed per housing unit [21]. Responses were taken from the Women’s Questionnaire. From these responses, women were selected to answer the Domestic Violence Module. This study conducted a secondary analysis using data from the 2022 P-DHS.

### Selection of study population

The primary inclusion criteria were female respondents 15–49 years old who had been sexually active and responded ‘yes’ or ‘no’ to having experienced an STI or related symptoms (genital ulcers and abnormal vaginal discharge) in the past year. The sample size was limited by the availability of responses for domestic violence and sexual autonomy. Respondents were either married or living with a partner. The final study sample size was 12178 females. The applied weights of the subsample were adjusted to the original dataset to conserve the sampling validity of the DHS with accurate regional representation.

The outcome is a composite of three variables: having an STI, presence of a genital ulcer, and abnormal genital discharge [22]. All occurrences were self-reported in the last 12 months from when the interview was conducted in May-June 2022.

### Investigated variables

Sociodemographic variables were included: age, urban vs. rural residence, highest educational level attained, wealth index categorized, occupation categorized, and religion. Frequency of alcohol consumption, and sexual history such as: age at first sexual contact, recent sexual activity, number of lifetime partners, and condom use were also investigated. Sexual autonomy was characterized by the ability to refuse sex, ask a partner to wear a condom, and a history of reproductive coercion. For IPV, having a controlling partner and a history of emotional, physical, and sexual abuse were included. Appendix A shows a description and a complete list of the variables included. The person deciding on the respondent’s healthcare, purchases, and family visits was also investigated: the respondent alone, their partner, relative, or others.

The respondent’s perception of domestic abuse justification (termed “wife beating justification” in the DHS) was characterized by a score based on five questions. Is a husband justified in beating his wife if: 1) she goes out without telling her husband, 2) neglects her children, 3) argues with her husband, 4) refuses to have sex with her husband, or 5) burns the food? Two points were given for ‘yes’ or in agreement, one for ‘I don’t know’ or uncertain, and zero for ‘no’ or disagreement. A higher score indicates that the respondent believes that physical domestic violence of a wife by her husband is justified in most of the given scenarios [23].

### Statistical methods used

Data analysis was conducted in R version 4.4.1. Descriptive statistics, Pearson’s chi-square test, Kruskal-Wallis test, and univariate modified Poisson regression analysis were performed to determine significant associations between variables and STI. A significant *p*-value of < 0.05 was used.

In building the multivariate regression model, variables were selected primarily based on literature and theoretical relevance [23–25]. For the modified Poisson regression analysis [26], *p*-values from a Wald-type hypothesis testing and link test for model misspecification were used, as well as the weighted area under the receiver operating curve (AUC-ROC), to guide model building. Wald-type hypothesis testing assessed the significance of the coefficients versus a zero coefficient. A *p*-value < 0.05 meant the null hypothesis could be rejected, and the combination of coefficients improved model fit [27]. The link test for generalized linear models was used to check for violated assumptions given a group of variables [28]. The weighted AUC-ROC measured the discriminative power of the model. The model estimated crude and adjusted risk ratios, along with their 95% confidence intervals. Multicollinearity was checked using the variance inflation factor (VIF). Variables with VIFs less than 5 were included.

## Results

Out of 12,178 respondents (unweighted count), STI prevalence among Filipino females was 6.96% (CI: 6.19%, 7.73%), with a weighted frequency of 1339. The total weighted count was 19,228. All answered the domestic violence module: currently married, cohabiting or separated from their husbands, or non-marital cohabiting unions.

Table 1 shows a cross-tabulation between sexual practices and perceptions, domestic violence experiences, and their STI outcome. *P*-values were from the Pearson’s chi-square test. Among the sociodemographic factors, only residence type was significantly associated with STI: more rural than urban cases. Women 20–24 years old had the greatest proportion of STI (10.11%). The proportion of STI cases was greater among those who experienced reproductive coercion (14.86%) versus those who had not experienced it (6.59%). Various types of domestic violence were significantly linked to STI: emotional, physical, and sexual. Fear of their current partner and abuse from a previous partner were also associated with an STI. Respondents’ ability to refuse sex, ask a partner to wear a condom, and recent condom use were notably not associated with STI in this population.

**Table 1:** Prevalence of STI by sociodemographic characteristic, sexual perceptions, practices, and intimate partner violence

Variable	STI			
	No	Yes	Weighted Frequency	*P*-value
Age in categories				0.128
15–19	91.09%	8.91%	378	
20–24	89.89%	10.11%	1633	
25–29	92.46%	7.54%	2926	
30–34	93.11%	6.89%	3702	
35–39	94.50%	5.50%	3598	
40–44	92.86%	7.14%	3707	
45–49	93.85%	6.15%	3284	
Residence type				**0.025**
Urban	93.95%	6.05%	10,424	
Rural	91.96%	8.04%	8804	
Highest education level attained				0.657
None	93.66%	6.34%	161	
Primary	93.56%	6.44%	2646	
Secondary	92.61%	7.39%	9510	
Tertiary	93.42%	6.58%	6910	
Wealth index categorized				0.655
Poorest	92.68%	7.32%	3846	
Poorer	92.12%	7.88%	3854	
Middle	93.20%	6.80%	4024	
Richer	93.86%	6.14%	3876	
Richest	93.34%	6.66%	3629	
Occupation categories				0.122
Unemployed	93.31%	6.69%	7881	
Professional/technical/managerial	93.54%	6.46%	2525	
Clerical	95.12%	4.88%	917	
Sales	91.04%	8.96%	3422	
Agricultural- self-employed	92.20%	7.80%	936	
Services	92.54%	7.46%	940	
Skilled manual	92.42%	7.58%	594	
Unskilled manual	94.56%	5.44%	1930	
Don’t know	95.47%	4.53%	85	
Religion				0.484
Roman Catholic	92.85%	7.15%	14,564	
Protestant	93.34%	6.66%	1858	
Iglesia ni Cristo	94.56%	5.44%	502	
Aglipay	91.55%	8.45%	287	
Islam	94.80%	5.20%	1358	
Other Christian	93.82%	6.18%	453	
No religion	93.05%	6.95%	20	
Other	88.41%	11.59%	187	
Recent sexual activity				**0.015**
In the last 4 weeks	92.64%	7.36%	14,227	
More than 4 weeks, postpartum	90.03%	9.97%	651	
More than 4 weeks, non-postpartum	94.79%	5.21%	4350	
Wife is justified to ask husband with STI to wear a condom ^a^				0.247
No	93.31%	6.69%	3473	
Yes	93.08%	6.92%	15,294	
I don’t know	89.73%	10.27%	461	
Not having sex because husband has other women				0.924
No	92.69%	7.31%	2036	
Yes	93.08%	6.92%	16,984	
I don’t know	92.85%	7.15%	208	
Can Refuse Sex				0.082
No	94.28%	5.72%	1434	
Yes	92.89%	7.11%	17,595	
I don’t know	97.46%	2.54%	199	
Can Ask Partner to Wear a Condom				0.115
No	92.35%	7.65%	3782	
Yes	93.08%	6.92%	14,716	
I don’t know	95.79%	4.21%	731	
Reproductive Coercion				**< 0.001**
No	93.41%	6.59%	18,372	
Yes	85.14%	14.86%	856	
Living with Partner				0.104
Yes	93.21%	6.79%	17,892	
No	90.71%	9.29%	1336	
Union status				0.755
Married	92.96%	7.04%	12,669	
Cohabitation	93.19%	6.81%	6559	
Condom used during last sex with most recent partner				0.311
No	92.91%	7.09%	17,812	
Yes	93.38%	6.62%	425	
No Answer	95.26%	4.74%	991	
Emotional violence ^a^				**< 0.001**
No	93.76%	6.24%	16,386	
Yes	88.91%	11.09%	2842	
Physical violence- less severe ^a^				**0.029**
No	93.21%	6.79%	18,072	
Yes	90.29%	9.71%	1156	
Physical violence- more severe ^a^				**0.004**
No	93.19%	6.81%	18,750	
Yes	87.25%	12.75%	478	
Sexual violence ^a^				**< 0.001**
No	93.26%	6.74%	18,835	
Yes	82.48%	17.52%	393	
Injuries from partner ^a^				**0.028**
No	93.17%	6.83%	18,636	
Yes	88.93%	11.07%	592	
Hurt partner unprovoked				**0.008**
No	93.30%	6.70%	17,785	
Yes	89.85%	10.15%	1443	
Partner consumes alcohol				0.667
No	93.23%	6.77%	8788	
Yes	92.88%	7.12%	10,440	
Respondent’s father ever beat mother				**0.021**
No	93.35%	6.65%	16,213	
Yes	91.03%	8.97%	2698	
I don’t know	94.17%	5.83%	317	
Coerced to perform unwanted sexual acts				**0.016**
No	93.13%	6.87%	18,929	
Yes	86.10%	13.90%	274	
Prefer not to answer	96.94%	3.06%	25	
Afraid of husband/partner				**< 0.001**
Never	93.64%	6.36%	15,760	
Most of the time	84.36%	15.64%	220	
Sometimes	90.69%	9.31%	3247	
Previous husband/partner abused respondent				**0.001**
Never	92.38%	7.62%	12,318	
< 1 year ago	98.73%	1.27%	39	
> 1 year ago	89.86%	10.14%	313	
Don’t remember when	88.75%	11.25%	454	
Never had another partner	94.81%	5.19%	6104	
No	93.37%	6.63%	11,441	
Yes	92.54%	7.46%	7787	
Person deciding respondent’s health care				**0.009**
Respondent alone	93.24%	6.76%	8902	
Respondent and partner	92.88%	7.12%	9332	
Husband/partner alone	93.00%	7.00%	985	
Someone else	100.00%	0.00%	7	
Other	0.00%	100.00%	4	
Person deciding respondent’s big purchases				**0.025**
Respondent alone	91.68%	8.32%	4068	
Respondent and partner	93.69%	6.31%	12,830	
Husband/partner alone	92.05%	7.95%	2278	
Someone else	79.22%	20.78%	33	
Other	87.57%	12.43%	18	
Person deciding respondent’s visits to family or relatives				**0.003**
Respondent alone	92.06%	7.94%	4067	
Respondent and husband/partner	93.49%	6.51%	13,676	
Husband/partner alone	91.91%	8.09%	1473	
Someone else	49.63%	50.37%	12	
Number of sex partners, excluding spouse, in last 12 months"				0.186
0	93.05%	6.95%	19,135	
1	91.54%	8.46%	92	
2	22.07%	77.93%	1	

*P*-values in bold indicate statistically significant results (*p < *0.05).

^***a***^Refer to Appendix A for full description of the variable

Table 2 presents the mean, median, and *p*-values for STI with continuous variables. Most variables are positively skewed. The median and interquartile range (IQR) were used since the data was not normally distributed; however, the difference in central tendency could not be observed with these alone, hence the mean and standard deviation (SD) were also included. Women with STI had partners with more controlling issues compared to non-infected women (*p*-value < 0.001). For a list of controlling issues, refer to Appendix A. The number of sexual partners in their lifetime, age at first sexual contact, number of non-spousal sexual partners, and frequency of alcohol consumption were not associated with STI in this population.

**Table 2:** Descriptive statistics of continuous variables for women with and without STI

	Range	Without STI				With STI				
		**Weighted Mean**	**SD**	**Weighted Median**	**IQR**	Weighted Mean	SD	Weighted Median	IQR	*p*-value
Justified domestic violence score ^a^	0–10	0.28	0.97	0	0	0.49	1.42	0	0	0.105
Age at first sexual contact	8–46	20.19	3.95	19	4	20.02	4.14	19	5	0.219
Number of controlling issues with partner ^a^	0–5	0.59	1.02	0	1	0.87	1.26	0	1	**< 0.001**
Days in a month consuming alcohol	0–30	0.35	1.58	0	0	0.48	2.35	0	0	0.618
Number of sexual partners in the last year	0–2	0.00	0.07	0	0	0.01	0.09	0	0	0.688
Total lifetime sexual partners	1–20	1.46	3.58	1	0	1.42	2.53	1	0	0.934

*P*-values in boldindicate statistically significant results (*p* < 0.05).

^***a***^Refer to Appendix A for full description of the variable

Table 3 shows the crude and adjusted estimated risk ratios from a modified Poisson regression analysis. Results shown are from one model only, so risk ratios of domestic violence variables are adjusted for sociodemographic factors and sexual perceptions and practices. *P*-values shown are for the adjusted risk ratios. Women who were 30–49 years old had a lower risk of STI compared to 20–24-year-olds. Muslims had lower STI risk compared to Catholics (ARR: 0.60, [CI: 0.38, 0.95]); this was the only significant finding among religion types. Consistent with bivariate analysis, reproductive coercion increased STI risk by 85%. Women who perceived domestic abuse to be justified had an increased risk of STI: a one-point increase in the score led to a 12% increase in STI risk. Emotional violence and fear of their partner consistently increased the risk for STI (ARR: 1.29 and 1.33, respectively).

**Table 3:** Risk ratios of STI by sexual factors and IPV in Filipino women

		CRR	95%CI	ARR	95%CI	*p*-value	
Age in Categories	15–19	0.88	[0.40,1.92]	0.87	[0.42,1.81]	0.716	
	20–24	REF		REF			
	25–29	0.75	[0.49,1.13]	0.73	[0.50,1.08]	0.115	
	30–34	0.68	[0.48,0.97]	0.61	[0.43,0.86]	0.005	**
	35–39	0.54	[0.39,0.76]	0.49	[0.35,0.68]	< 0.001	***
	40–44	0.71	[0.50,1.00]	0.65	[0.45,0.94]	0.023	*
	45–49	0.61	[0.39,0.94]	0.57	[0.36,0.91]	0.019	*
Residence Type	Urban	REF		REF			
	Rural	1.33	[1.03, 1,71]	1.25	[0.97,1.61]	0.078	.
Education level	None	REF		REF			
	Primary	1.02	[0.34,3.05]	0.79	[0.27,2.30]	0.667	
	Secondary	1.17	[0.39,3.48]	0.92	[0.31,2.73]	0.882	
	Tertiary	1.04	[0.35,3.11]	0.85	[0.27,2.70]	0.788	
Wealth index	Poorest	REF		REF			
	Poorer	1.08	[0.84,1.38]	1.04	[0.80,1.35]	0.792	
	Middle	0.93	[0.67,1.29]	0.95	[0.70,1.30]	0.770	
	Richer	0.84	[0.60,1.18]	0.92	[0.58,1.43]	0.698	
	Richest	0.91	[0.63,1.31]	1.03	[0.68,1.55]	0.889	
Occupation categories	Unemployed	REF		REF			
	Professional/technical/managerial	0.97	[0.72,1.29]	1.00	[0.73,1.37]	0.996	
	Clerical	0.73	[0.47,1.14]	0.78	[0.49,1.24]	0.285	
	Sales	1.34	[0.99,1.81]	1.32	[0.97,1.78]	0.074	.
	Agricultural- self-employed	1.17	[0.83,1.63]	1.13	[0.80,1.60]	0.483	
	Services	1.11	[0.71,1.75]	1.06	[0.69,1.64]	0.786	
	Skilled manual	1.13	[0.66,1.94]	1.13	[0.65,1.95]	0.674	
	Unskilled manual	0.81	[0.59,1.12]	0.82	[0.60,1.10]	0.187	
	Don’t know	0.68	[0.26,1.79]	0.60	[0.22,1.63]	0.316	
Religion	Roman Catholic	REF		REF			
	Protestant	0.93	[0.67,1.29]	0.85	[0.63,1.14]	0.285	
	Iglesia ni Cristo	0.76	[0.38,1.53]	0.72	[0.35,1.50]	0.384	
	Aglipay	1.18	[0.68,2.06]	1.06	[0.63,1.79]	0.824	
	Islam	0.73	[0.48,1.10]	0.60	[0.38,0.95]	0.029	*
	Other Christian	0.86	[0.46,1.61]	0.94	[0.51,1.71]	0.830	
	No religion	0.97	[0.20,4.80]	1.60	[0.33,7.80]	0.561	
	Other	1.62	[0.83,3.15]	1.44	[0.71,2.92]	0.313	
Days consumed alcohol monthly ^***b***^		1.03	[0.99, 1.07]	1.02	[0.98,1.06]	0.380	
Age at first sexual contact ^***b***^		0.99	[0.96, 1.02]	1.02	[0.99,1.05]	0.295	
Current marital status	Married	REF		REF			
	Unmarried, living with a partner	0.97	[0.79, 1.19]	0.70	[0.55,0.88]	0.002	**
Residing with partner	Yes	REF		REF			
	No	1.37	[0.94, 1.99]	1.62	[1.10,2.36]	0.013	*
Recent sexual activity	In the last 4 weeks	REF		REF			
	More than 4 weeks, postpartum	1.35	[0.85,2.15]	1.32	[0.87,1.99]	0.186	
	More than 4 weeks, non-postpartum	0.71	[0.53,0.94]	0.66	[0.49,0.90]	0.010	**
Justified to ask partner with STI to wear a condom	No	REF		REF			
	Yes	1.03	[0.80,1.33]	1.12	[0.86,1.45]	0.407	
	I don’t know	1.53	[0.98,2.39]	1.72	[1.11,2.66]	0.014	*
Number of sex partners, excluding spouse, in last 12 months	0	REF		REF			
	1	1.22	[0.35, 4.24]	1.01	[0.30,3.42]	0.982	
	2	11.21	[6.01, 20.91]	2.89	[1.19,6.99]	0.019	*
Total lifetime sexual partners ^***b***^		1.00	[0.98, 1.01]	0.99	[0.97,1.01]	0.343	
Not having sex because husband has other women	No	REF		REF			
	Yes	0.95	[0.70,1.27]	0.93	[0.67,1.30]	0.682	
	I don’t know	0.98	[0.41,2.30]	0.86	[0.41,1.81]	0.692	
Respondent can refuse sex	No	REF		REF			
	Yes	1.24	[0.89,1.74]	1.45	[1.00,2.12]	0.051	.
	I don’t know	0.44	[0.15,1.34]	0.70	[0.21,2.36]	0.561	
Respondent can ask partner to use a condom	No	REF		REF			
	Yes	0.90	[0.74,1.11]	0.93	[0.76,1.14]	0.475	
	I don’t know	0.55	[0.31,0.99]	0.58	[0.31,1.11]	0.100	
Condom used during last sex with most recent partner	No	REF		REF			
	Yes	0.93	[0.47,1.85]	0.98	[0.49,1.96]	0.949	
	No response	0.67	[0.40,1.12]	0.60	[0.35,1.03]	0.062	.
Justified domestic violence score ^a, ***b***^		1.14	[1.06, 1.23]	1.12	[1.04,1.21]	0.004	**
Reproductive coercion	No	REF		REF			
	Yes	2.25	[1.64, 3.09]	1.85	[1.37,2.49]	< 0.001	***
Number of controlling issues from partner ^a, ***b***^		1.22	[1.13, 1.31]	1.07	[0.98,1.18]	0.129	
Emotional violence ^a^	No	REF		REF			
	Yes	1.78	[1.44, 2.19]	1.29	[1.01,1.65]	0.039	*
Physical violence- less severe ^a^	No	REF		REF			
	Yes	1.43	[1.04, 1.97]	0.78	[0.51,1.20]	0.257	
Physical violence- more severe ^a^	No	REF		REF			
	Yes	1.87	[1.22, 2.86]	1.16	[0.66,2.04]	0.600	
Sexual violence ^a^	No	REF		REF			
	Yes	2.60	[1.68, 4.02]	1.47	[0.97,2.22]	0.069	.
Injuries from partner ^a^	No	REF		REF			
	Yes	1.62	[1.06, 2.48]	0.97	[0.52,1.84]	0.935	
Hurt partner unprovoked	No	REF		REF			
	Yes	1.51	[1.12, 2.05]	1.17	[0.85,1.61]	0.328	
Partner consumes alcohol	No	REF		REF			
	Yes	1.05	[0.84, 1.32]	0.87	[0.67,1.12]	0.270	
Respondent’s father ever beat mother	No	REF		REF			
	Yes	1.35	[1.07,1.70]	1.14	[0.91,1.44]	0.248	
	I don’t know	0.88	[0.46,1.69]	0.72	[0.39,1.30]	0.272	
Coerced to perform unwanted sexual acts	No	REF		REF			
	Yes	2.03	[1.15,3.57]	1.46	[0.88,2.41]	0.138	
	Prefer not to answer	0.45	[0.06,3.32]	0.34	[0.04,2.81]	0.316	
Respondent afraid of partner	Never	REF		REF			
	Most of the time	2.46	[1.43,4.24]	1.49	[0.81,2.74]	0.200	
	Sometimes	1.46	[1.15,1.87]	1.33	[1.03,1.70]	0.026	*
Respondent hit by previous partner	Never	REF		REF			
	< 1 year ago	0.17	[0.02,1.30]	0.09	[0.01,0.76]	0.027	*
	> 1 year ago	1.33	[0.73,2.41]	1.15	[0.68,1.92]	0.603	
	Don’t remember when	1.48	[0.86,2.53]	1.50	[0.91,2.47]	0.109	
	Never had another partner	0.68	[0.55,0.85]	0.65	[0.52,0.80]	< 0.001	***
Person deciding on respondent’s health care	Respondent alone	REF		REF			
	Respondent and partner	1.05	[0.84,1.32]	1.24	[1.00,1.54]	0.055	.
	Husband/partner alone	1.04	[0.69,1.56]	0.96	[0.60,1.53]	0.863	
	Someone else	< 0.01	< 0.01	< 0.01	[< 0.01, < 0.01]	< 0.001	***
	Other	14.79	[12.79,17.10]	6.08	[1.04,35.35]	0.045	*
Person deciding on respondent’s large household purchases	Respondent alone	REF		REF			
	Respondent and partner	0.76	[0.60,0.96]	0.83	[0.66,1.05]	0.122	
	Husband/partner alone	0.96	[0.70,1.30]	0.97	[0.70,1.35]	0.846	
	Someone else	2.50	[0.86,7.29]	0.93	[0.31,2.82]	0.897	
	Other	1.49	[0.24,9.42]	0.97	[0.22,4.22]	0.972	
Person deciding on respondent’s visits to family or relatives	Respondent alone	REF		REF			
	Respondent and husband/partner	0.82	[0.64,1.04]	0.92	[0.73,1.17]	0.510	
	Husband/partner alone	1.02	[0.68,1.52]	1.10	[0.71,1.69]	0.675	
	Someone else	6.34	[2.84,14.19]	3.26	[0.97,11.00]	0.057	.

*P*-value equal or less than: *0.05, **0.005, ***0.0005, ‘.’ 0.1

^***a***^Refer to Appendix A for a full description of the variable

^***b***^Variables were analyzed as continuous variables

Respondents who were uncertain about the acceptability of requesting condom use from a partner with STI had a greater risk of STI compared to those who did not feel it was warranted at all (ARR: 1.72 [CI: 1.11, 2.66]). Women who could refuse sex also had a borderline increased risk of STI (ARR: 1.45, [CI: 1.00, 2.12]). For union status, women living separately from their partners had a higher risk of STI. Unmarried women living with their partners had a lower risk of STI compared to married women. This is evident after correcting for sociodemographic factors and domestic violence. Women who had been in previous unions without a history of domestic abuse had a higher risk of STI as well. Having two non-spousal sexual partners in the past 12 months nearly tripled the risk of STI; however, the confidence interval was wide (ARR: 2.89 [CI: 1.19, 6.99]) with few respondents.

## Discussion

Emotional abuse, fear of one’s partner, and reproductive coercion among Filipino women increased their risk of STI. Physical, sexual abuse, and having controlling partners were also linked to STI. A related study found Filipino women in abusive relationships had lower odds of requesting condom use from their partner [23]. IPV victims are at risk for STI complications with lower rates of cervical cancer screening; despite slightly higher rates of screening among Filipino IPV victims, these suggest awareness of sexual health risks in their relationships [29–31]. Women coerced into pregnancy reported partners threatening to leave them for using birth control, contributing to fear around condom negotiation [13, 32]. The relationship between STI and IPV is not always linear. A history of STI increased women’s risk of being in another abusive relationship due to feelings of worthlessness from a past infection; the same feelings made women susceptible to coerced sexual situations, increasing STI risk [33]. In this current study, STI risk increased by 12% when women justified IPV. A related study found that women who believed IPV to be acceptable had lower odds of reproductive health-seeking behavior [34]. Justifying IPV can hinder women from leaving abusive relationships, prolonging their exposure to an increased risk of STI.

Recent condom use, number of sexual partners, and age of first sexual contact were not associated with STI in this population. This study does not negate them as established factors that directly affect STI transmission [15, 25]. Instead, it points to other concerning and influential factors, such as power imbalance in relationships. The temporality of condom use was also not specified in the DHS: whether used before or after contracting an STI. Respondents’ ability to refuse sex increased the risk of STI in this study. Although counterintuitive, other studies show that sexual autonomy and awareness may increase willingness to report and get tested for STI [35, 36]. Uncertainty in asking a partner with an STI to wear a condom increased STI risk. This could also reflect more frequent STI testing among respondents doubtful of their partner’s status. However, there may be inadequate understanding of STI prevention as well since general condom negotiation was not significantly linked to STI. The hesitancy arose when their partner had an STI. Studies found that the lack of sex education in conservative Muslim societies may increase STI risk [6]. However, the lower risk found in this study compared to Catholics could mean muslims are also getting tested less or simply uncomfortable in disclosing their STI history. The variables indicating who decides on the respondent’s healthcare, purchases, and family visits were not strongly associated with STI prevalence. This suggested that not all forms of agency are equally linked to STI risk. Gender dynamics in intimate relationships may be more influential. Lower STI risk among women recently abused by a former partner versus those never abused may not necessarily be meaningful due to the relatively few responses (Tables 1 and 3). Subgroup analysis would provide a clearer picture of its link with STI since studies in other countries have opposing results [37, 38]. Respondents having other people deciding for their healthcare had lower STI risks (Tables 1 and 3) but also had few responses. These other people may be more knowledgeable and effective in making health-related decisions. This finding would be more conclusive with subgroup analysis as well.

The alarming prevalence of domestic abuse justification across education and income levels demands an inquiry into early values formation in basic human rights. Reproductive health counseling should be more transparent in how harmful attitudes can increase STI risk among women. This way, the population will not take these beliefs lightly, knowing certain health outcomes depend on it. School-based sex education may not always be reliable, with inadequate reproductive health pedagogy from educators [39]. Although not all religions were associated with STI in this study, many oppose reproductive health education policies, hindering healthy conversations around sexuality [39]. Conflicted followers make reproductive health implementation and STI control difficult. Instead of complete opposition, sex education can align with some religious values formation, such as abstinence, but also how and why to say no. This develops negotiating skills that strengthen sexual autonomy, which can lower the odds of STI [40]. Another crucial point of intervention is medical visits. Healthcare practitioners in clinics, HIV treatment hubs, and health centers catering to women’s health do not always have the time and training to assess IPV and sexual history appropriately. However, this study shows its importance in STI surveillance. IPV victims with an STI history would also require more than contraceptive use counseling, given the trauma, abusive living situation, and relationship dynamics linked with it. Aside from expanding routine STI testing to IPV survivors, correcting values and perceptions underlying their intimate relationships is crucial in STI control. Patients must be aware and intentional of how values clarification exercises and motivational and cognitive-behavioral elements cultivate healthy sexual practices [40].

This study calls for STI prevention counseling tailored for domestic violence victims. Sexual consent and negotiation skills must be adequately and appropriately integrated into adolescent sex education to prevent victims of domestic violence and STI. Although this study focused on a vulnerable female population, male aggression and the unconditional acceptance of their dominance in households contribute to pervasive abuse. Public health interventions of STI control must be founded on strong values for human rights in both men and women.

### Strengths and limitations of the study

Although the sample was representative of each region in the country, responses for the domestic violence module were limited to women who had been married or cohabited with a partner. Self-reporting STI could have underestimated the prevalence. Aggregating STI symptoms with STI history may have overestimated the frequency since abnormal discharge and genital ulcers are nonspecific symptoms. It also was not determined how respondents knew of their STI diagnosis: whether they got tested or simply suspected. Recall bias may have been present, given that the data were collected through a health survey. However, this study was a significant step in identifying STI prevalence on a national scale, beyond the known high-risk groups. It established the added danger of STI among women in abusive relationships.

Temporality of condom use and STI treatment were not specified in the DHS. This study did not include women not in unions or currently having more than two sexual partners. Perceptions of justified domestic abuse of wives by their husbands are assumed to be the respondent’s own opinion. Whether it reflects their husband’s or family’s opinion cannot be determined through the dataset. The number of non-spousal sexual partners and persons making decisions for the respondent may have significant p-values, but weighted frequencies are too small to be conclusive. Although some sexual practices were not consistently associated with STI, they may be contributory factors, perhaps in subgroups of women. This study however, was still able to characterize women at risk of STI in the population.

## Conclusion

STI prevalence is linked with IPV and women believing it can be justified. Having a controlling partner and experiencing coercion make it difficult for women to voice their sexual preferences, putting them at risk for STI. This study demonstrates the need for routine STI testing among IPV victims. Reinforcing health centers’ capacity and prompt referral from the Barangay Violence Against Women (VAW) desk would therefore be required. This study also advises including sexual perceptions, practices, living situations, and history of abuse and coercion in STI risk assessment to strengthen STI surveillance. Supplementary training is essential for frontline health workers, who frequently serve as initial responders to IPV survivors.

Compulsory pre-marriage family planning seminars should emphasize human rights and sexual autonomy. Reinforcing these in seminars promotes gender equality and reproductive rights among couples. It is also well within the mandate of the Reproductive Health Law to ensure a rights-based approach as a foundation of sex education. Instilling the values of gender equality, respect, and consent must be strictly implemented regardless of educators’ beliefs. This study shows that women’s harmful attitudes of justifying IPV can increase STI risk. Cultivating women empowerment is therefore crucial in STI prevention, by reducing women’s susceptibility to coercion, imbalanced gender dynamics, and IPV.

## Appendix

**Table:** 

Variable	Description
Age in 5-year Groups	15–19, 20–24, 25–29, 30–34, 35–39, 40–44, 45–49
Residence Type	Urban, rural
Education	Highest attained: none, primary, secondary, tertiary
Wealth Index Category	Poorest, poorer, middle, richer, richest
Occupation (Grouped)	Not working, Professional/technical/managerial, Clerical, Sales, Agricultural - self-employed, Agricultural - employee, Household and domestic, Services, Skilled manual, Unskilled manual, Don’t know
Religion	Roman Catholic, Protestant, Iglesia ni Cristo, Aglipay, Islam, Other Christian (not otherwise categorizable), No religion, Other; not specified if practicing or not
Current marital status	Married, living with partner
Currently residing with husband/partner	Living with partner, staying elsewhere
Number of days respondent drank alcoholic drinks in the past month	Continuous variable; range: 0–30
Age at first sexual contact	Continuous age variable
Recent Sexual Activity	Active in last 4 weeks, Not active in last 4 weeks - postpartum abstinence, not active in last 4 weeks - not postpartum abstinence; Sexual acts not specified
Wife is justified in asking husband to use condom if he has STI	No, yes, don’t know. Respondents’ perception if asking a partner with STI to wear a condom is warranted
Total lifetime number of sex partners	Continuous variable; range: 1–20
Number of sex partners, excluding spouse, in last 12 months	Categorized: 0, 1, 2
Reason for not having sex: husband has other women	No, yes, don’t know
Respondent can refuse sex	No, yes, don’t know
Respondent can ask partner to use a condom	No, yes, don’t know
Husband or family member pressured respondent to become pregnant	No, yes
Currently residing with husband/partner	Living with partner, staying elsewhere
Justified domestic abuse score	Respondent’s perception that physical beating of a wife by her husband is justified if wife: goes out without telling husband, neglects the children, argues with husband, refuses to have sex with husband, burns the food; belief that domestic abuse of a wife by her husband is justified in given situations; 0- no (disagree), 1- I don’t know (uncertain), 2- yes (agree). Summed up into a score, continuous variable, range: 0–10.
Number of control issues experienced	Husband/partner: jealous if respondent talks with other men, accuses respondent of unfaithfulness, does not permit respondent to meet female friends, tries to limit respondent’s contact with family, insists on knowing where respondent is; 0- no, 1-yes; summed up (range: 0–5)
Experienced any emotional violence	Ever been humiliated by husband/partner, threatened with harm by husband/partner, insulted or made to feel bad by husband/partner, not allowed to engage in any legitimate work, have no control your own money or properties or forces you to work, husband/partner destroy your personal properties, pets or belongings; no, yes (any one of these).
Experienced any less severe violence by husband/partner	Ever been pushed, shook or had something thrown by husband/partner, been slapped by husband/partner, punched with fist or hit by something harmful by husband/partner, had arm twisted or hair pulled by husband/partner; no, yes (any one of these).
Experienced any severe violence by husband/partner	Ever been kicked or dragged by husband/partner, strangled or burnt by husband/partner, been threatened with knife/gun or another weapon by husband/partner; no, yes (any one of these).
Experienced any sexual violence by husband/partner	Ever been physically forced into unwanted sex by husband/partner. been forced into other unwanted sexual acts by husband/partner, been physically forced to perform sexual acts respondent didn’t want to; no, yes (any one of these).
Experienced any of these actions from husband/partner	Ever had bruises because of husband/partner’s actions, had eye injuries, sprains, dislocations or burns because of husband/partner, had wounds, broken bones, broken teeth or other serious injury; no, yes (any one of these).
Respondent ever physically hurt husband/partner when he was not hurting her	No, yes
Husband/partner drinks alcohol	No, yes
Respondent’s father ever beat her mother	No, yes, don’t know
Coerced to perform unwanted sexual acts	No, yes, refused to answer/no response. Specific sexual acts not specified
Respondent afraid of husband/partner most of the time, sometimes or never	Never, most of the time, sometimes
Previous husband: ever hit, slap, kick or physically hurt respondent	Never, less than a year ago, more than a year ago, yes but don’t remember, never had another husband/partner
Person who usually decides on: respondent’s health care”	Respondent alone, respondent and partner, partner alone, someone else, other
Person who usually decides on: large household purchases”	Respondent alone, respondent and partner, partner alone, someone else, other
Person who usually decides on: visits to family or relatives”	Respondent alone, respondent and partner, partner alone, someone else, other

## Data Availability

Data is publicly available upon request at the DHS website: https://dhsprogram.com/data/dataset/Philippines_Standard-DHS_2022.cfm?flag=1.

## Abbreviations

ARR: Adjusted Risk Ratio
CI: Confidence Intervals
CRR: Crude Risk Ratio
(P)DHS: (Philippine) Demographic Health Survey
FSW: Female Sex Worker
HIV: Human Immunodeficiency Virus
IPV: Intimate Partner Violence
IQR: Interquartile Range
MSM: Men having Sex with Men
SD: Standard Deviation
STI(s): Sexually Transmitted Infection(s)
USA: United States of America
WHO: World Health Organization

## Footnotes

1
https://dhsprogram.com/data/dataset/Philippines_Standard-DHS_2022.cfm?flag=1.
